# Association of rs2282679 polymorphism in vitamin D binding protein gene (GC) with the risk of vitamin D deficiency in an iranian population: season-specific vitamin D status

**DOI:** 10.1186/s12902-023-01463-7

**Published:** 2023-10-10

**Authors:** Golaleh Asghari, Emad Yuzbashian, Leila Najd-Hassan-Bonab, Parvin Mirmiran, Maryam S. Daneshpour, Fereidoun Azizi

**Affiliations:** 1 Nutrition and Endocrine Research Center, Research Institute for Endocrine Sciences, Shahid Beheshti University of Medical Sciences, Tehran, Iran; 2 Cellular and Molecular Endocrine Research Center, Research Institute for Endocrine Sciences, Shahid Beheshti University of Medical Sciences, Tehran, 19195-4763 Iran; 3 Endocrine Research Center, Research Institute for Endocrine Sciences, Shahid Beheshti University of Medical Sciences, Tehran, Iran

**Keywords:** Vitamin D, 25-hydroxyvitamin D, rs2282679

## Abstract

**Background:**

Genome-wide association studies in Western countries indicate a considerable impact of variations in vitamin D binding protein (GC) genes on serum concentrations of 25-hydroxyvitamin D (25(OH)D). We aimed to investigate an association between rs2282679 polymorphism in GC and vitamin D deficiency.

**Methods:**

A cross-sectional study conducted in the framework of the Tehran Cardio-Metabolic Genetic Study (TCGS) cohort. A total of 1568 participants aged > 18 years were randomly selected, and their 25(OH) D concentration was measured. Vitamin D deficiency was assessed concerning rs2282679 by descriptive and multivariate analysis, odds ratio (OR), and 95% confidence intervals (95%CI) calculated. Since the interaction term between rs2282679 and recruitment season was significant, we performed regression analysis separately for individuals whose blood was taken in high sunny and those whose blood was drawn in the low sunny season.

**Results:**

The rs2282679 polymorphism was in Hardy-Weinberg equilibrium (P > 0.05) in the studied population. The serum concentration of 25(OH) D median was 15.0 ng/mL, and the prevalence of VDD was 27.8%. The presence of the G allele in rs2282679 increases the risk of VDD in additive (OR = 1.35, 95% CI: 1.06–1.73) and dominant (OR = 1.33, 95% CI: 1.06–1.68) genetic models. After separating participants based on the recruitment season, the unfavorable association was observed in the additive and dominant only in the low sunny season.

**Conclusion:**

The finding of the current study indicates that the GC rs2282679 SNP is associated with vitamin D deficiency. It seems that the impact of risk allele increased in the low sunny season when UV exposure has been declined.

## Introduction

Vitamin D status, evaluated by 25-hydroxyvitamin D (25(OH)D) concentration, has been identified not only as a vital element for musculoskeletal health, but also as an important factor concerning the development of non-communicable diseases including metabolic syndrome, type 1, and 2 diabetes, hypertension, cardiovascular disease, colon, breast, and prostate cancer [1]. There is disagreement as to which value serum 25(OH)D level might be optimal; however, most of the evidence indicated that vitamin D deficiency (VDD) or severe deficiency is defined as values below 10 ng/mL (25 nmol/L) [2, 3]. Applying this definition to VDD was strongly associated with an increased risk of several health-related outcomes [4, 5]. Season, age, sex, obesity, and smoking were previously reported to be associated with VDD [5, 6]. However, genetic determinants of VDD are less understood, especially in the Iranian population.

Genetic variations that are engaged in vitamin D transport, metabolism, and synthesis are responsible for inter-individual differences in vitamin D concentration [7–9]. In this regard, genome-wide association studies (GWAS) identified common polymorphisms, single nucleotide polymorphism (SNP) such as vitamin D binding protein correlated with serum concentrations of 25(OH)D [10]. Vitamin D binding protein, which is encoded by the group-specific component (GC) gene and synthesized by the liver, is a circulating protein that binds to 25(OH)D and transports it in the circulation, which, in turn, affects the bioavailability of active 25(OH)D [11]. In humans, the GC gene located at chromosome 4 (4q13.3) and recent GWAS among different ethnicities have indicated that the rs2282679 T > G in the GC gene has robustly linked to VDD [10, 12–15]. Notably, the presence of the G allele in this SNP is associated with decreased 25(OH) D concentration and a higher risk of VDD [10, 12, 13]. However, these studies are mostly conducted in Western and Eastern Asian populations [10, 12, 13, 16, 17]. No study has yet evaluated the association of this genetic variant with VDD in the Iranian population. In this cross-sectional study, we aimed to assess the association of genetic variants in GC (rs2282679) with VDD in the Tehran Cardio-metabolic Genetic Study (TCGS).

## Method

### Study population

This cross-sectional study was conducted within the framework of the Tehran Cardiometabolic Genetic Study (TCGS). TCGS is part of an ongoing family-based cohort study, the Tehran lipid, and glucose study (TLGS). TLGS, as a parent study to TCGS, is a prospective cohort study being conducted on a representative sample of residents of District No. 13 of Tehran, the capital of Iran, (35.7˚ N), as part of Shahid Beheshti University of Medical Sciences and Health Services coverage, to determine the prevalence and incidence of non-communicable diseases (NCD) and related risk factors. Detailed information concerning TLGS and TCGS, as described previously [18, 19].

**Fig. 1:**
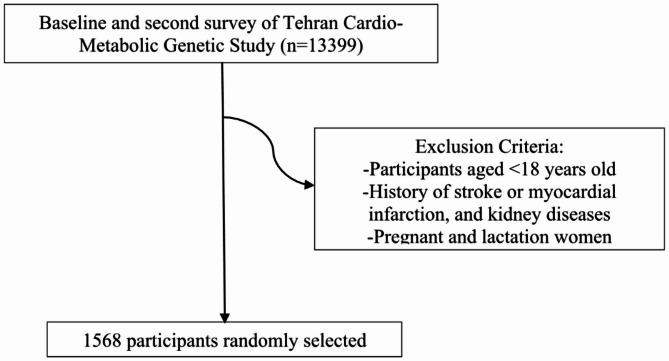
Flow chart of participants included in the present study

The current study was based on data from the baseline and the second survey of 13,399 individuals in TCGS. We excluded those < 18 years of age, with a history of stroke or myocardial infarction, kidney diseases, pregnant or lactating. From the remaining individuals, a subsample of 1568 participants, recruited from winter, spring, summer, and fall, was randomly selected for 25(OH)D measurements (Fig. 1). Informed written consent was obtained from all participants. The ethics committee of the Research Institute for Endocrine Sciences approved the study. All methods of the current study were performed following the relevant guidelines and regulations.

### Vitamin D and other covariate measurements

Blood samples were drawn after overnight fasting of 12–14 h to evaluate 25(OH) D concentration and some other metabolic risk factors, including the fasting plasma glucose (FPG), triglyceride (TG), and cholesterol concentration using the enzymatic colorimetric method. Serum 25(OH)D was measured by the enzyme immunoassay (EIA) method (Immunodiagnostic System Ltd, Boldon, UK). The intra and inter-assay coefficients of variation (% CV) were 2.7 and 3.9, respectively. In the current study, the value of 25(OH)D < 10ng/mL was the cutoff value for the diagnosis of VDD [20, 21].

Participants completed a questionnaire, including demographic and clinical characteristics such as age, sex, marital status, education level, medication use, smoking, and family history of chronic diseases. Waist circumference (WC), weight, and height were measured based on the standard protocols [18], and body mass index (BMI) was calculated as weight (kilogram) divided by the square of height (meter). Systolic and diastolic blood pressure were obtained by the mean of two measurements taken on the right arm at an interval of 5 min.

### GC genotype assessment

#### Genotyping

Samples were washed with lysis buffer where PBS and RBCs were separated. DNA was extracted from the WBCs with the alkaline boiling method and stored at -20°C. By spectrophotometry and electrophoresis, quantitative and qualitative assessments of the extracted DNA were performed. DNA samples of 13,399 TCGS participants were genotyped with Illumina Human OmniExpress-24-v1-0 bead chip containing 649,932 SNP loci at the deCODE genetics company (Iceland) according to manufacturer’s specifications (Illumina Inc., San Diego, CA, USA) [19]. After quality control, the rs2282679 genotype was extracted from the chip-typed data for all individuals.

### Statistical analysis

Variable distributions were evaluated by the Kolmogorov-Smirnov test and histogram chart. The descriptive results were expressed as mean (standard deviation (SD) for normally distributed variables, as median (25–75 interquartile range (IQR)) for variables with a skewed distribution, or percentage for categorical variables. Skewed variables were log-transformed for statistical analysis. Student’s t-test and chi-square (χ^2^) test were applied to compare sociodemographic and clinical variables between deficient and sufficient vitamin D participants. Participants’ characteristics were also examined using ANOVA according to the categories of genotype. VDD was defined as serum levels of 25(OH)D less than 10 ng/mL [20, 21].

Multivariable logistic regression models were applied to evaluate the association of SNP with the prevalence of VDD after adjusting for the following covariates: age (continuous), sex (male and female), BMI (continuous), season (summer and fall as high sunny seasons and winter and spring as low sunny seasons), and smoking status (current and former or never).

If the alleles of the gene of interest are T and G in haploid, and G is the ‘increasing’ or ‘risk’ allele, the one causing an effect, the three genotype groups would then be TT, TG, and GG. This dichotomization of the SNP genotypes can be done as follows: Additive: ‘TT’ *versus* ‘TG’ *versus* ‘GG’. By creating interaction terms of SNP in the additive model (0 for homozygous for the non-risk allele, 1 for heterozygous, and 2 for homozygous for the risk allele) with age (aged less or higher than 60 years), sex (male and female), obesity status (non-obese and obese), season (high sunny season and fall and low sunny season), and smoking status (current and former or never), we tested whether these variables affected the relationship between genotypes and VDD. If the interaction terms were significant, participants would be stratified according to that variable.

With the final model, a sensitivity analysis was performed to address the possibility that outliers for 25(OH) D may affect the final results. Therefore, we excluded participants whose 25(OH) D concentration was ± 1.5 IQR.

## Results

The rs2282679 polymorphism was in Hardy-Weinberg equilibrium (P > 0.05). Overall, the genotype frequency was 7.7% and 53.8% for homozygous GG and TT, respectively, and 38.5% for heterozygous GT. The median serum concentration of 25(OH) D was 15.0 ng/mL, and the prevalence of VDD was 27.8%. The average (SD) age was 48.6 (13.2) years, and BMI was 27.9 (4.4) kg/m^2^. Approximately 50% of the participants were women, and 29% were smokers. The median 25(OH) D concentration of sufficient individuals was 20.0 (IQR: 13.6–30.9) ng/mL, while those who were insufficient had a median 25(OH) D concentration of 7.6 (IQR: 5.9-9.0) ng/mL. Given the status of vitamin D, participants with a deficiency were younger, were more likely to be female, and had a higher BMI and cholesterol concentration and a lower frequency of smoker in comparison to those who were vitamin D sufficient (Table 1).

**Table 1:** Characteristics of study participants according to the vitamin D deficiency

	Total	Vitamin D sufficient (> 10 ng/ml)	Vitamin D deficient (< 10 ng/ml)	p-value
Age (year)	48.6 (13.2)	49.2 (13.3)	47.1 (13.1)	0.007
Female (%)	50.6	42.6	71.3	< 0.001
Body mass index (kg/m^2^)	27.9 (4.4)	27.7 (4.4)	28.3 (4.5)	0.031
Fasting plasma glucose (mg/dl)	99.9 (33.5)	99.2 (29.9)	101.6 (41.3)	0.200
Triglycerides (mg/dl)	148.0 (105.0-217.0)	149.0 (105.0-217.0)	148.0 (105.0-216.7)	0.849
Cholesterol (mg/dl)	212.7 (44.2)	211.0 (42.6)	216.9 (47.9)	0.019
Smoker (%)	29.1	31.8	22.0	< 0.001
Recruitment season (%)				
Spring	25.6	23.5	31.0	< 0.001
Summer	28.0	30.8	20.6	
Fall	26.4	28.4	21.1	
Winter	20.0	17.2	27.3	
25-hydroxy vitamin D (ng/ml)	15.0 (9.95–25.3)	20.0 (13.6–30.9)	7.6 (5.9-9.0)	< 0.001

Data are presented as mean (SD) or median (25–75 percentile) for quantitative variables according to their distribution and percent for categorical variables

Across the rs2282679 polymorphism, each additional risk allele was associated with an increased prevalence of VDD to 25.2, 30.8, and 30.6%, respectively (p = 0.049). There was no significant difference between the three groups regarding the clinical and demographical characteristics of participants (Table 2).

**Table 2:** Characteristics of study participants according to the GC single nucleotide polymorphism rs2282679 genotypes^1,2^

	TT (n = 844)	GT (n = 603)	GG (n = 121)	p-value
Age (year)	48.2 (13.3)	49.1 (13.2)	48.6 (13.4)	0.521
Female (%)	50.7	49.4	55.4	0.486
Body mass index (kg/m^2^)	27.8(4.5)	27.9(4.2)	28.3(4.7)	0.542
Fasting plasma glucose (mg/dl)	98.5(32.9)	101.5(34.8)	101.2(29.9)	0.230
Triglyceride (mg/dl)	148 (105–216)	146 (105–213)	162 (105–235)	0.771
Cholesterol (mg/dl)	213 (44.3)	212 (44.1)	216 (44.3)	0.573
Smoker (%)	29.3	29.9	24.0	0.423
Season of recruitment (%)				
Spring	24.8	26.0	28.9	0.196
Summer	30.1	25.9	24.0	
Fall	24.2	28.7	30.6	
Winter	21.0	19.4	16.5	
Vitamin D deficiency (%)	25.2	30.8	30.6	0.049
25-hydroxy vitamin D (ng/ml)	15.5(10–26)	14.0(9.5–25.5)	13.6(8.9–25.8)	0.328

^1^Data are presented as mean (SD) or median (25–75 percentile) for quantitative variables according to their distribution and percent for categorical variables

^2^ The alleles of GC rs2282679 are T and G in haploid, and G is the ‘risk’ allele, therefore, the three genotype groups would then be TT, TG and GG.

The rs2282679 T > G polymorphism was associated with increasing the risk of VDD in additive and dominant genetic models. In the additive model the adjusted OR for participants with heterozygous genotype compared to those with TT genotype was 1.35 (95% CI: 1.06–1.73). In the dominant model, the adjusted OR for participants with the GT + GG genotype compared to those with the TT genotype was 1.33 (95% CI: 1.06–1.68). There was no significant association of GC SNP rs2282679 with VDD in the recessive model (Table 3).

**Table 3:** Odds ratio (OR) and 95% confidence interval (CI) for the association of GC single nucleotide polymorphism rs2282679 with vitamin D deficiency

	Cases/total	Crude OR (95% CI)	p-value	Adjusted OR (95% CI)^1^	p-value
Additive					
TT (Reference)	213/844	1.00		1.00	
GT	186/603	1.32 (1.04–1.67)	0.019	1.35 (1.06–1.73)	0.015
GG	37/121	1.30 (0.86–1.98)	0.211	1.24 (0.80–1.93)	0.332
Dominant					
TT	213/844	1.00		1.00	
GT + GG	223/724	1.32 (1.06–1.64)	0.014	1.33 (1.06–1.68)	0.015
Recessive					
TT + GT	399/1447	1.00		1.00	
GG	37/121	1.16 (0.77–1.73)	0.479	1.09(0.71–1.67)	0.687

^1^Adjusted for age, sex, body mass index, smoking, and recruitment season

Since there was a significant interaction between recruitment season and rs2282679 on VDD, we performed logistic regression analysis separately for those whose blood was collected in high and low sunny seasons to evaluate if these associations changed with season. In the low sunny season, participants who carried one or two copies of the risk allele had a substantially higher risk for VDD than those with homozygous for the common allele (OR = 1.63, 95% CI: 1.15–2.30, and OR = 1.92, 95% CI: 1.06–3.47, respectively). In the dominant model, there was an increased risk of VDD for participants with the GT + GG genotype compared to those with the common allele group (OR = 1.67, 95% CI: 1.20–2.33). However, no relationship existed between VDD and rs2282679 SNP variants (Table 4).

**Table 4:** Odds ratio (OR) and 95% confidence interval (CI) for the association of GC single nucleotide polymorphism rs2282679 with vitamin D deficiency according to high and low sunny season recruitment

	High sunny season				Low sunny season		
	Cases/total	Adjusted OR (95% CI)	p-value		Cases/total	Adjusted OR (95% CI)^1^	p-value
Additive							
TT (Reference)	121/463	1.00			92/381	1.00	
GT	89/313	1.13 (0.80–1.60)	0.481		94/290	1.63 (1.15–2.30)	0.006
GG	15/64	0.58 (0.42–1.61)	0.579		22/57	1.92 (1.06–3.47)	0.032
Dominant							
TT	121/463	1.00			92/381	1.00	
GT + GG	104/377	1.08 (0.77–1.50)	0.658		119/347	1.67 (1.20–2.33)	0.002
Recessive							
TT + GT	210/776	1.00			189/971	1.00	
GG	15/64	0.78 (0.41–1.51)	0.471		22/57	1.54 (0.87–2.12)	0.139

^1^Adjusted for age, sex, body mass index, and smoking status

In the sensitivity analyses, after excluding participants with 25(OH) D concentration was ± 1.5 IQR, no substantial impact was observed. The adjusted ORs for additive and dominant models were 1.52 (95% CI: 1.17–1.98) and 1.74 (95% CI: 1.24–2.45), respectively, when blood was drawn in the low sunny season (data not shown).

## Discussion

In this population-based study of individuals living in Tehran, there was a direct association between the numbers of G alleles in rs2282679 and the prevalence of VDD. In the additive model, we observed a 35% as well as 24% increased risk of VDD for the presence of each G allele in heterozygote and homozygote rs2282679 genotypes. Dominant models for the risk allele also had remarkable support for a 33% increased risk of VDD in participants with the GT + GG genotype compared to those with the TT genotype. These relationships were confounded by season so that the association between genotypes of rs2282679 and increased risk of VDD was more robust in the low sunny season and disappeared in the high sunny season.

The impact of rs2282679 located GC, encoding vitamin D-binding protein, which transports vitamin D metabolites to target tissues, is reported in several studies in Western populations and East Asia. Consistent with our findings, Brouwer-Brolsma et al. reported that the presence of G allele rs2282679 SNP was strongly associated with 25(OH)D status among Dutch older adults [22]. Furthermore, the GWAS study indicated that per copy of the risk G allele had a 50% increased risk for VDD among Caucasians [10]. Inconsistent with our findings, Li et al. illustrated that the risk alleles of GC rs2282679 SNP had no significant association with VDD among 1199 participants from China [17]. Also, the Danish population with the minor G allele showed slightly higher serum 25(OH)D levels than those with the T allele [23]. The existing G allele in this SNP was associated with lower concentrations of vitamin D binding protein, which leads to lower bioavailability of 25(OH)D to target organs [24].

**Fig. 2:**
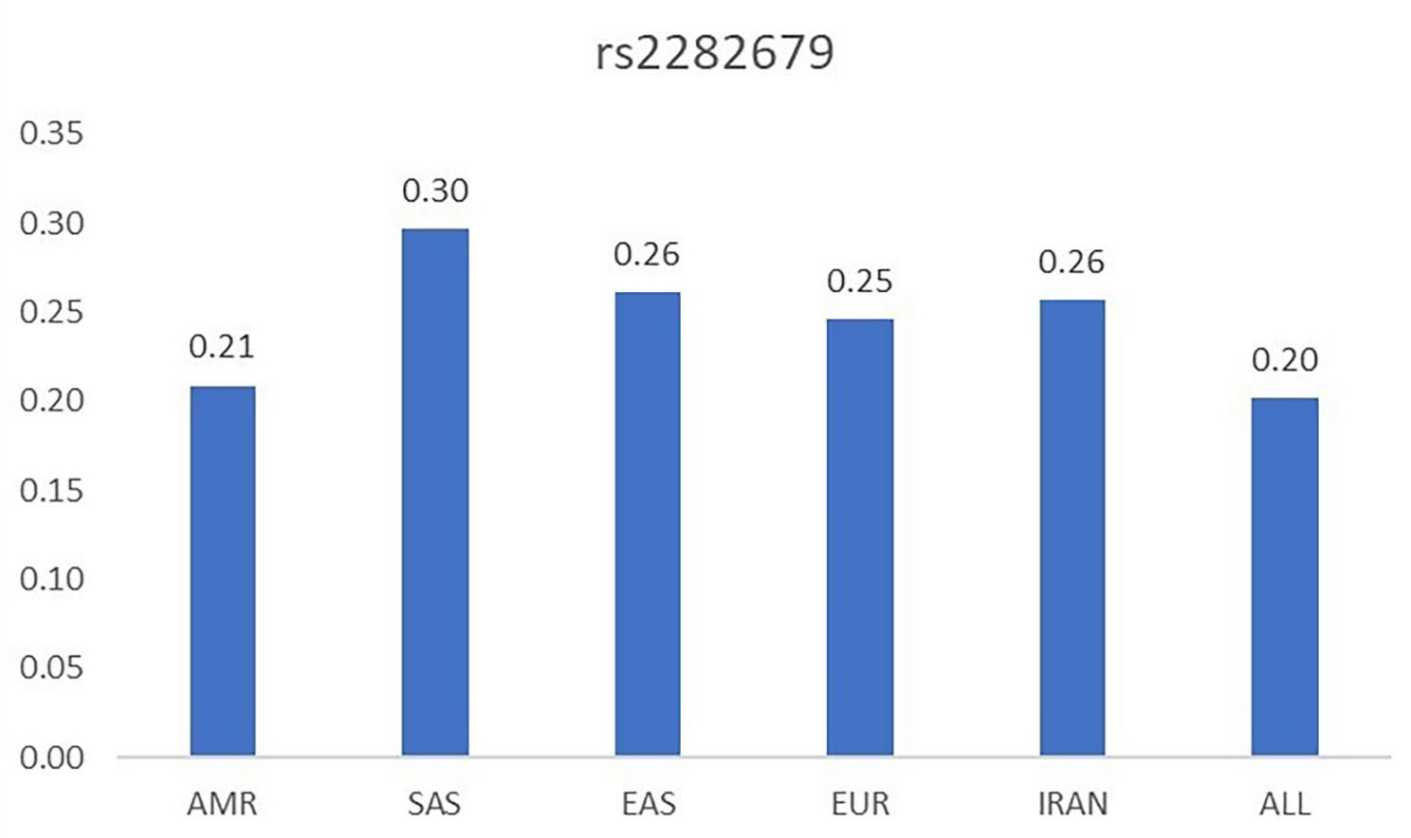
Comparison of minor allele frequency (MAF) in different populations

In our community-based study with a wide range of ages between 18 and 72 years, to better understand the relationship between variation in rs2282679 and VDD, we examined the interaction of age, sex, obesity status, season, and smoking status with assessed genotype on the prevalence of VDD. We found a significant interaction for the season of drawing blood. In contrast to our finding, Perna et al. illustrated that the inverse associations between the risk allele of rs2282679 with 25(OH)D concentration among older adults were more robust from May to October and disappeared from November to April [12]. Furthermore, another study reported that per copy of the G allele, the association of rs2282679 SNP with 25(OH)D status was stronger during summer/autumn [22]. The discrepancy of the impact of season on the presence of G allele with vitamin deficiency might be explained by the prevalence of the risk allele in those studies in comparison with us. As Fig. 2 shows (https://www.ensembl.org/index.html?redirect=no), the minor allele frequency (MAF) distribution of the rs2282679 in Iran and five continents. Although the MAF distribution of the rs2282679 among different countries has no considerable variation, the prevalence of minor alleles in our study sample was comparable to that in other study populations. We found that the risk allele in our population became widespread, and its prevalence is around 54%; however, the prevalence of risk alleles in both samples of the population was 38% in the study of Perna et al., [12] 8% in the study of Brouwer-Brolsma et al. [22]. Since an admixed population such as Iranian residence consisted of various ethnic populations with hidden relationships and consanguineous marriages, sophisticated admixtures in the population may cause analytical risks that measure the association of loci with the risk of developing a disease population with different profiles, especially in those undergoing admixture.

Although the underlying mechanism of action remains unclear, it seems that during low sunny seasons, winter and spring, the human body relies more on its vitamin D_3_ reserves than during the high sunny seasons, summer and fall, that the body produces vitamin D_3_ endogenously from 7-dehydrocholesterol when ultraviolet (UV) rays from sunlight strike the skin and trigger vitamin D synthesis [25]. In this regard, Holic et al. reported that the body uses vitamin D3 reserves in body fat during the winter month when vitamin D3 cannot be produced [26]. Accordingly, it can be assumed that the individuals genetic susceptibilities relating to vitamin D metabolism pathways in regulating vitamin D status during low sunny seasons, may be further attributed to genes that transmit vitamin D throughout the body, such as rs2282679 polymorphism in the GC gene, which have a more colorful role than other genes related to vitamin D metabolism pathways. While, through the summer and fall with higher sun exposure, the skin becomes the primary source of vitamin D synthesis, which may decrease the effect modification of gene variation relating to vitamin D transporting throughout the body [27]. Interestingly, according to Holick’s rule stated that sun exposure 1/4 of a minimal erythemal dose over 1/4 of a body produces an adequate amount of vitamin D (1000 IU) [28]. It is important to mention that the body produces vitamin D from sun exposure, when the UV index, which is the level of the sun’s UV radiation measurement method proposed by the world health organization and it ranges from zero upward, is above 3 [29]. Furthermore, Abdi et al. demonstrated that the mean UV index value in Iran were 10.56, 3.4, 3.24, and 8.65 in summer, fall, winter, and spring, respectively [30]. Therefore, it seems that the UV index in Iran during all seasons is sufficient for vitamin D production. It should be noted that there are additional factors to be considered, including skin color, sunscreen use, wearing protective clothing, less time spent outdoors, and air pollution, which reduces the amount of body exposure to sunlight.

It is believed that sun exposure leads to vitamin D creation in the skin, and then to synthesize hepatic form i.e. 25(OH) vitamin D. The 25(OH) vitamin D is carried by vitamin D binding-protein in the bloodstream. The genotype effect of rs6013897 on the degradation of 25(OH)D becomes seemingly more evident when 25(OH)D concentrations are higher (> 30 ng/mL) for a longer period of time. Despite that rs6013897 is also widely recognized to catabolize 1,25-dihydroxyvitamin D [1,25(OH)2D], the active vitamin D metabolite, the genotype effect of rs6013897 on 1,25(OH)2D seems to have an improbable impact on circulating levels of serum 25(OH)D [31].

Examined Swedish twins indicated that genetic impact explained 50% of the variability of 25(OH)D in summer, while the environment largely explained the variance in winter [32]. Moreover, Nissen et al. illustrated that the genotype effects of CYP2R1 and GC SNPs on 25(OH)D status do not vary concerning vitamin D source, neither diet nor UVB exposure [23]. Therefore, the influence of sun exposure on 25(OH)D status could be modified by several SNPs.

One of the reasons of this direct relationship might be the dependency of the translation of GC on UV-B radiation. Furthermore, DHCR7 encoding for the enzyme 7-dehydrocholesterol reductase having the role in synthesis of cholesterol in skin, so that there is no 7-dehydrocholesterol metabolized into vitamin D [22]. Hence, the hypothesis is that DHCR7 that we did not examine, might be encoded in low sunny season leading to VDD.

The first and most vital strength of our research is that important confounders related to vitamin D concentration such as age, sex, obesity, smoking status, and season and also their interactions were considered. Likewise, the studied population was from TCGS, and SNP subsets were selected based on GWAS. However, some limitations should be considered to interpret the findings of the current study. The cross-sectional design of the study does not allow the determination of causal relationships. The considered SNP were chosen from previous reports conducted in China, the USA, and European populations. Furthermore, we evaluated only one genetic variant on the GC gene; thus, the exclusion of polymorphisms in other genes with a contribution to the vitamin D metabolism pathway would not be possible. Although the demographic characteristics of participants in the current study were homogenous, middle-aged urban adults, these results may not be generalizable to other ethnicities or age groups. The further limitation of our study was the lacking of data on vitamin D intake via diet or supplements, use of sunscreen, wearing protective clothing, and spending less time engaged in outdoor activities.

## Conclusions

Our data demonstrated that the GC rs2282679 is associated with vitamin D deficiency. It seems that the impact of risk allele increased in the low sunny season when UV exposure has been declined. Due to the fact that one size does not fit all, ensuring adequate vitamin D levels may require a tailored approach considering the rs2282679 in GC genotypes, especially in individuals with G allele, particularly in the low sunny season and also further randomized clinical trials are required to approve these findings.

## Data Availability

The datasets generated and/or analyzed during the current study are available in the [TCGS] repository, [http://www.gemiran.org/rs2282679.html].

## Abbreviations

VDD: Vitamin D Deficiency
GWAS: genome-wide association studies
GC: the group-specific component
TLGS: Tehran Lipid and Glucose Tolerance
TCGS: Tehran Cardio-metabolic Genetic Study
NCD: non-communicable diseases
FPG: fasting plasma glucose
TG: triglyceride
EIA: enzyme immunoassay
WC: waist circumference
BMI: body mass index
SD: standard deviation
IQR: interquartile range
SNP: single nucleotide polymorphism
MAF: the minor allele frequency

## References

[1] Wolden-Kirk H, Gysemans C, Verstuyf A, Mathieu C (2012). Extraskeletal effects of vitamin D. Endocrinol Metabolism Clin.

[2] McKenna M, Freaney R (1998). Secondary hyperparathyroidism in the elderly: means to defining hypovitaminosis D. Osteoporos Int.

[3] Lavie CJ, Lee JH, Milani RV (2011). Vitamin D and cardiovascular disease: will it live up to its hype?. J Am Coll Cardiol.

[4] Pilz S, Marz W, Wellnitz B, Seelhorst U, Fahrleitner-Pammer A, Dimai HP, Boehm BO, Dobnig H (2008). Association of vitamin D deficiency with heart failure and sudden cardiac death in a large cross-sectional study of patients referred for coronary angiography. J Clin Endocrinol Metab.

[5] Lee JH, O’Keefe JH, Bell D, Hensrud DD, Holick MF (2008). Vitamin D deficiency: an important, common, and easily treatable cardiovascular risk factor?. J Am Coll Cardiol.

[6] Chan J, Jaceldo-Siegl K, Fraser GE (2010). Determinants of serum 25 hydroxyvitamin D levels in a nationwide cohort of blacks and non-hispanic whites. Cancer Causes Control.

[7] Sinotte M, Diorio C, Berube S, Pollak M, Brisson J (2009). Genetic polymorphisms of the vitamin D binding protein and plasma concentrations of 25-hydroxyvitamin D in premenopausal women. Am J Clin Nutr.

[8] Engelman CD, Fingerlin TE, Langefeld CD, Hicks PJ, Rich SS, Wagenknecht LE, Bowden DW, Norris JM (2008). Genetic and environmental determinants of 25-hydroxyvitamin D and 1, 25-dihydroxyvitamin D levels in hispanic and african Americans. J Clin Endocrinol Metabolism.

[9] Xu W, Sun J, Wang W, Wang X, Jiang Y, Huang W, Zheng X, Wang Q, Ning Z, Pei Y (2014). Association of genetic variants of vit D binding protein (DBP/GC) and of the enzyme catalyzing its 25-hydroxylation (DCYP2R1) and serum vit D in postmenopausal women. Horm (Athens).

[10] Wang TJ, Zhang F, Richards JB, Kestenbaum B, Van Meurs JB, Berry D, Kiel DP, Streeten EA, Ohlsson C, Koller DL (2010). Common genetic determinants of vitamin D insufficiency: a genome-wide association study. The Lancet.

[11] Powe CE, Ricciardi C, Berg AH, Erdenesanaa D, Collerone G, Ankers E, Wenger J, Karumanchi SA, Thadhani R, Bhan I (2011). Vitamin D–binding protein modifies the vitamin D–bone mineral density relationship. J Bone Miner Res.

[12] Perna L, Felix JF, Breitling LP, Haug U, Raum E, Burwinkel B, Schöttker B, Brenner H. Genetic variations in the vitamin D binding protein and season-specific levels of vitamin D among older adults. Epidemiology 2013:104–9. 10.1097/EDE.0b013e318276c4b0 23191998

[13] Ahn J, Yu K, Stolzenberg-Solomon R, Simon KC, McCullough ML, Gallicchio L, Jacobs EJ, Ascherio A, Helzlsouer K, Jacobs KB (2010). Genome-wide association study of circulating vitamin D levels. Hum Mol Genet.

[14] Rivera-Paredez B, Macías N, Martínez-Aguilar MM, Hidalgo-Bravo A, Flores M, Quezada-Sánchez AD, Denova-Gutiérrez E. Association between vitamin D Deficiency and single nucleotide polymorphisms in the vitamin D receptor and GC genes and analysis of their distribution in mexican postmenopausal women. Nutrients. 2018. 10. 10.3390/nu10091175 PMC6164456 30150596

[15] Santos BR, Costa NC, Silva TR, Oppermann K, Magalhães JA, Casanova G, Spritzer PM (2019). Prevalence of vitamin D deficiency in women from southern Brazil and association with vitamin D-binding protein levels and GC-DBP gene polymorphisms. PLoS ONE.

[16] Slater NA, Rager ML, Havrda DE, Harralson AF (2017). Genetic variation in CYP2R1 and GC genes associated with vitamin D deficiency status. J Pharm Pract.

[17] Li L-H, Yin X-Y, Wu X-H, Zhang L, Pan S-Y, Zheng Z-J, Wang J-G (2014). Serum 25 (OH) D and vitamin D status in relation to VDR, GC and CYP2R1 variants in chinese. Endocr J.

[18] Azizi F, Ghanbarian A, Momenan AA, Hadaegh F, Mirmiran P, Hedayati M, Mehrabi Y, Zahedi-Asl S (2009). Prevention of non-communicable disease in a population in nutrition transition: Tehran lipid and glucose study phase II. Trials.

[19] Daneshpour MS, Fallah M-S, Sedaghati-Khayat B, Guity K, Khalili D, Hedayati M, Ebrahimi A, Hajsheikholeslami F, Mirmiran P, Tehrani FR (2017). Rationale and design of a genetic study on cardiometabolic risk factors: protocol for the Tehran Cardiometabolic Genetic Study (TCGS). JMIR Res Protocols.

[20] Vitamin D. for bones [https://theros.org.uk/information-and-support/bone-health/vitamin-d-for-bones/?_gl=1*5ojmj5*_up*MQ&gclid=Cj0KCQjw_r6hBhDdARIsAMIDhV8d_vvXmHTrfGUbB3u0QAHZgsmNBMhGF0zVb-TWM4vrKHJixA0oCFoaAoLcEALw_wcB].

[21] Thacher TD, Clarke BL (2011). Vitamin D insufficiency. Mayo Clin Proc.

[22] Brouwer-Brolsma EM, Vaes AMM, van der Zwaluw NL, van Wijngaarden JP, Swart KMA, Ham AC, van Dijk SC, Enneman AW, Sohl E, van Schoor NM (2016). Relative importance of summer sun exposure, vitamin D intake, and genes to vitamin D status in Dutch older adults: the B-PROOF study. J Steroid Biochem Mol Biol.

[23] Nissen J, Vogel U, Ravn-Haren G, Andersen EW, Madsen KH, Nexø BA, Andersen R, Mejborn H, Bjerrum PJ, Rasmussen LB (2015). Common variants in CYP2R1 and GC genes are both determinants of serum 25-hydroxyvitamin D concentrations after UVB irradiation and after consumption of vitamin D3–fortified bread and milk during winter in Denmark. Am J Clin Nutr.

[24] Zhang Z, He JW, Fu WZ, Zhang CQ, Zhang ZL (2013). An analysis of the association between the vitamin D pathway and serum 25-hydroxyvitamin D levels in a healthy chinese population. J Bone Miner Res.

[25] Holick MF (2006). High prevalence of vitamin D inadequacy and implications for health. Mayo Clin Proc.

[26] Holick MF (2007). Vitamin D Deficiency. N Engl J Med.

[27] Rosenstreich SJ, Rich C, Volwiler W (1971). Deposition in and release of vitamin D 3 from body fat: evidence for a storage site in the rat. J Clin Investig.

[28] Dowdy JC, Sayre RM, Holick MF (2010). Holick’s rule and vitamin D from sunlight. J Steroid Biochem Mol Biol.

[29] How to Use the Sun Safely to Make Vitamin. D and Benefit Your Health [https://www.grassrootshealth.net/blog/use-sun-safely-make-vitamin-d-benefit-health/.

[30] Abdi M, Azimi Pirsaraei SR, Mohammadizadeh MR (2021). Evaluation of solar ultraviolet radiation in Zanjan city using the ultraviolet index. ijhe.

[31] Kwak S-Y, Park CY, Jo G, Kim OY, Shin M-J. Association among genetic variants in the vitamin D pathway and circulating 25-hydroxyvitamin D levels in korean adults: results from the Korea National Health and Nutrition Examination Survey 2011–2012. Endocr J 2018:EJ18–0084. 10.1507/endocrj.EJ18-0084 29937467

[32] Snellman G, Melhus H, Gedeborg R, Olofsson S, Wolk A, Pedersen NL, Michaëlsson K (2009). Seasonal genetic influence on serum 25-hydroxyvitamin D levels: a twin study. PLoS ONE.

